# Analysis of intervention effects of three different models on children with autism spectrum disorder

**DOI:** 10.3389/fpsyt.2026.1856696

**Published:** 2026-07-21

**Authors:** Lanqiu Lv, Liang Xu, Jiaqi Sun, Yanli Hu, Zenghe Yue, Yingbo Lv, Xiaoyan Ke

**Affiliations:** 1 Child Healthcare Department, Ningbo University Affiliated Women and Children Hospital, Ningbo, China; 2 Child Mental Health Research Center, The Affiliated Brain Hospital of Nanjing Medical University, Nanjing, China; 3 Tiantai County Maternal and Child Health Care and Family Planning Service Center, Taizhou, Zhejiang, China; 4 Nanjing Medical University, Nanjing, China

**Keywords:** autism spectrum disorder, family intervention, family-institutional intervention, institutional intervention, intervention effects

## Abstract

**Background:**

This study primarily investigates the intervention effects of different models on children with autism.

**Methods:**

A total of 132 autistic children were recruited and assigned to three intervention models: institutional intervention (N=45), family intervention (N=45), and family-institutional intervention (N=42). Comparisons were made of CARS scores before and after intervention across the three groups, as well as developmental quotients in the Gesell Developmental Schedules domains including adaptive behavior, gross motor skills, fine motor skills, language, and personal-social skills.

**Results:**

The results indicate that all three intervention models alleviated autistic symptoms in children post-intervention. Notably, the combined family-institutional intervention group demonstrated significant improvements in adaptive abilities, fine motor skills, language, and personal-social relationships.

**Conclusions:**

These findings suggest that while all three models effectively improved ASD symptoms, the combined family-institutional intervention was associated with more comprehensive improvements across key developmental domains.

## 1 Introduction

Autism Spectrum Disorder (ASD), commonly referred to as autism, is a group of neurodevelopmental disorders characterized primarily by social communication impairments and repetitive, stereotyped behaviors (1). The global incidence of ASD has shown a rising trend year by year, with an estimated prevalence of approximately 1% (2). According to the latest report from the US CDC, the prevalence of ASD is 2.76% (3), while reported prevalence in China is about 0.7% (4). ASD has become a significant global public health issue. Onset typically occurs in early childhood, and core symptoms persist throughout life. ASD is often accompanied by intellectual and behavioral challenges, which require substantial family commitment and societal support resources (5), and is one of the leading causes of childhood psychiatric disability both in China and globally (6, 7).

To date, the etiology of ASD remains unclear, and there are no specific pharmacological treatments. However, comprehensive interventions can promote the development of social communication skills, communication abilities, reduce repetitive behaviors, enhance self-care skills, and decrease maladaptive behaviors in children with ASD. With appropriate training, some individuals can achieve the ability to live, learn, and work independently in adulthood, thereby optimizing long-term outcomes for individuals and their families (8, 9).

Regarding intervention, the primary treatment for ASD focuses on education and training. Numerous specialized intervention models have been developed, such as Applied Behavior Analysis (ABA), Developmental Theories, and Naturalistic Developmental Behavioral Interventions (NDBI). While these methods target different domains and vary in effectiveness, they share common core elements and characteristics, notably emphasizing the crucial importance of family involvement in the intervention process. The home environment is the earliest and most significant environment a child encounters. Consequently, parent-mediated family interventions may offer distinct advantages for ASD rehabilitation (10). Family intervention refers to an approach where trained parents implement the intervention within the natural environment, aiming to improve social functioning and reduce maladaptive behaviors in individuals with ASD (11). In 2017, family intervention was formally recognized as an evidence-based practice (12).

However, many parents adopt a rejecting or wait-and-see attitude towards ASD intervention, often due to a lack of relevant knowledge, financial constraints, and frequently missing the critical window for early rehabilitation. This suggests potential issues with the accessibility and dissemination of family interventions (13). In recent years, ASD intervention approaches have increasingly emphasized naturalistic and diversified teaching methods, placed greater focus on leveraging parental participation and the involvement of typically developing peers, and increasingly prioritized building on children’s strengths. Therefore, there is an urgent need to identify more direct, cost-effective, and efficient intervention models to achieve effective treatment outcomes.

In this context, “early intervention” in this study is operationally defined as structured rehabilitation services initiated before the age of 6 years, targeting the critical period of neuroplasticity to maximize developmental gains. The primary objective of this study is to compare the therapeutic efficacy of three distinct models for children with ASD: (1) institution intervention (conventional professional rehabilitation training), (2) family intervention, and (3) a combined family-institution intervention model. By systematically analyzing the strengths, limitations, and clinical value of each modality, this study aims to optimize early rehabilitation strategies. Furthermore, we will evaluate the cost-effectiveness of these models to provide evidence for selecting direct, efficient, and sustainable intervention pathways that promote effective outcomes for children with autism.

## 2 Methods

### 2.1 Participants and procedures

This prospective non-randomized blinded controlled study enrolled a cohort of 132 children diagnosed with Autism Spectrum Disorder (ASD) at the Child Health Clinic of Ningbo Women and Children’s Hospital between June 2023 and November 2024. The study protocol was approved by the Ethical Review Board of Ningbo Women and Children’s Hospital and was pre-registered on 30 June 2021 via the Zhejiang Province Medical Research Project Registry (Registration No. 2022KY1156).

Inclusion criteria were: (1) aged 18 to 54 months; (2) meeting DSM-5 criteria for ASD confirmed by two senior pediatricians; (3) Childhood Autism Rating Scale (CARS) score≥30; (4) no severe sensory impairments or serious organic diseases. Exclusion criteria included: (1) comorbid severe psychiatric disorders; (2) known genetic syndromes (e.g., Fragile X) or history of traumatic brain injury; (3) prior participation in formal, structured rehabilitation programs. All participants were newly diagnosed within one month prior to enrollment and had not received systematic intervention previously.

Caregivers selected one of three intervention models: institutional intervention(Group 1), family intervention (Group 2), or combined family-institutional intervention (Group 3).

Group 1 (institutional intervention group): Received therapist-delivered structured teaching, sensory integration, and cognitive training (5 days/week, 5 hours total daily duration, 6 months) without parental participation during sessions.

Group 2 (family intervention group): Primary caregivers completed an eight-session parent training curriculum over two months, delivered by two certified therapists with 5 years of experience and specific qualifications in ASD. Caregivers then implemented strategies at home for 6 months (25 hours/week).

Group 3 (combined family-institutional intervention): This group received both institutional intervention (5 days/week, 3 hours total daily duration) and family intervention components concurrently over 6 months (10 hours/week).

**Figure 1:**
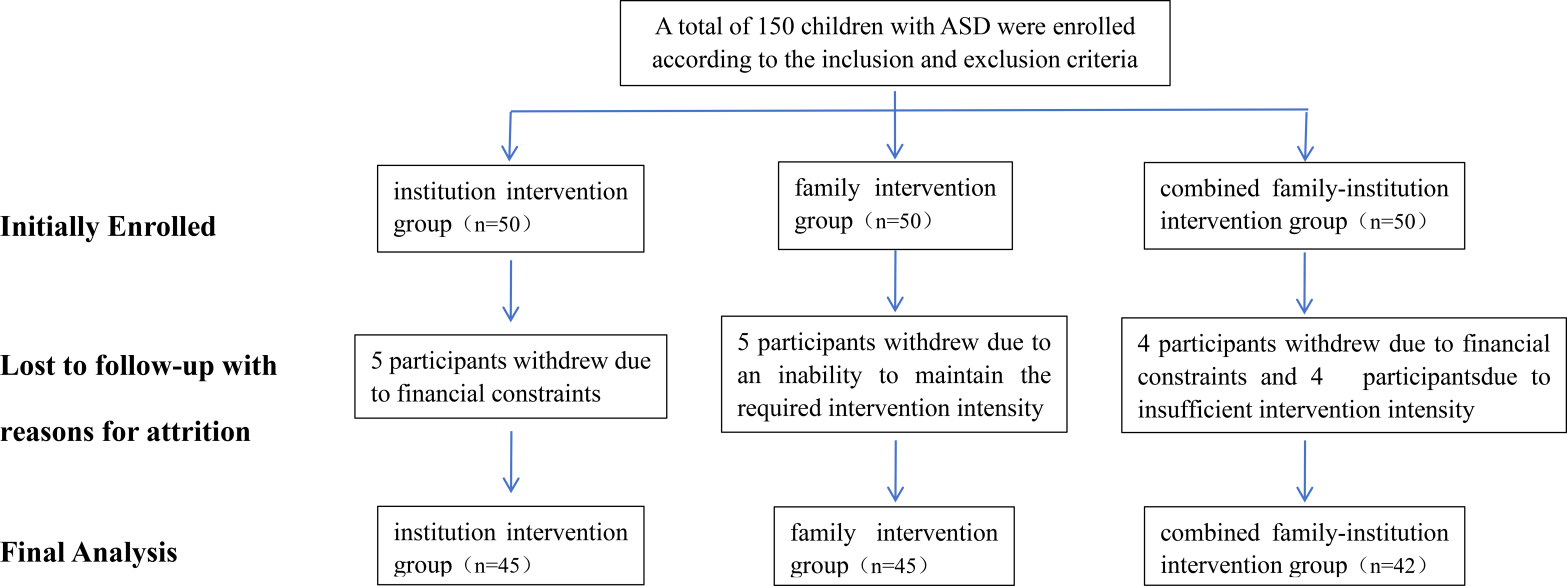
The participant flow diagram.

Adherence and Follow-up: for Groups 2 and 3, adherence was monitored through weekly video submissions, daily logbooks, and bi-weekly therapist feedback. During the final four-month maintenance phase, families continued independent practice, supported by monthly telephone follow-ups and a single mid-point booster session. Of the 150 participants initially enrolled, 18 were lost to follow-up, yielding an attrition rate of 12% (18/150). A total of 132 participants completed the study and were included in the final analysis (Group 1: n = 45; Group 2: n = 45; Group 3: n = 42). The reasons for attrition were as follows: in the institution intervention group (Group 1), 5 participants withdrew due to financial constraints; in the family intervention group (Group 2), 5 withdrew due to an inability to maintain the required intervention intensity; and in the combined family-institution intervention group (Group 3), 4 withdrew due to financial constraints and 4 due to insufficient intervention intensity. (**Figure 1**) Notably, all 18 families who discontinued the study were provided with tailored follow-up intervention plans.

Baseline demographic data were collected retrospectively via questionnaire. The collected variables included age, sex, place of residence, singleton status, parental age at childbirth, parental education level, birth order, parity, gestational age at delivery, and mode of delivery (**Table 1**).

**Table 1:** Comparison of baseline characteristics among the three groups.

Characteristic	Institutional intervention (*N* = 45)	Family intervention (*N* = 45)	Combined family-institutional intervention (*N* = 42)	*χ^2^*	*P*
Age in months	36.60 ± 10.56	36.40 ± 10.23	37.24 ± 10.25	*F* = 0.077	0.926
Gender (%)					
Male	37 (82.2)	36 (80.0)	33 (78.6)	0.187	0.911
Female	8 (17.8)	9 (20.0)	9 (21.4)		
Residence (%)					
Urban	35 (77.8)	39 (86.7)	32 (76.2)	1.783	0.410
Rural	10 (22.2)	6 (13.3)	10 (23.8)		
Maternal age at delivery (%)					
<35years	31 (68.9)	33 (73.3)	33 (78.6)	1.046	0.593
≥35years	14 (31.1)	12 (26.7)	9 (21.4)		
Paternal age at delivery (%)					
<35years	27 (60.0)	28 (62.2)	28 (66.7)	0.426	0.808
≥35years	18 (40.0)	17 (37.8)	14 (33.3)		
Maternal education (%)					
Primary/middle school	12 (26.7)	12 (26.7)	15 (35.7)	2.029	0.730
High school	11 (24.4)	9 (20.0)	6 (14.3)		
College/Graduate or higher	22 (48.9)	24 (53.3)	21 (50.0)		
Paternal education (%)					
Primary/middle school	11 (24.4)	13 (28.9)	11 (26.2)	7.530	0.110
High school	10 (22.2)	2 (4.4)	10 (23.8)		
College/Graduate or higher	24 (53.3)	30 (66.7)	21 (50.0)		
Gravidity (%)					
1	18 (40.0)	22 (48.9)	17 (40.5)	5.299	0.258
2	23 (51.1)	16 (35.6)	15 (35.7)		
≥3	4 (8.9)	7 (15.6)	10 (23.8)		
Parity (%)					
1	24 (53.3)	27 (60.0)	21 (50.0)	1.099	0.894
2	18 (40.0)	15 (33.3)	17 (40.5)		
≥3	3 (6.7)	3 (6.7)	4 (9.5)		
Gestational age (GA) (%)					
Preterm delivery	4 (8.9)	5 (13.3)	3 (7.1)	1.556	0.817
Term delivery	40 (88.9)	37 (82.2)	38 (90.5)		
Post-term delivery	1 (2.2)	2 (4.4)	1 (2.4)		
Delivery method					
Vaginal	25 (55.6)	33 (73.3)	28 (66.7)	3.194	0.202
Cesarean	20 (44.4)	12 (26.7)	14 (33.3)		
The severity of the illness (%)					
Mild-moderate	25 (55.6)	26 (57.8)	23 (54.8)	0.087	0.957
Severe	20 (44.4)	19 (42.2)	19 (45.2)		

Continuous variables were compared using one-way ANOVA (F value), and categorical variables were compared using Chi-square test (*χ²*). P values are two-tailed. ^***^p < 0.001, ^**^p < 0.01, ^*^p < 0.05.

Pre-intervention assessments using the Childhood Autism Rating Scale (CARS) and the Gesell Developmental Schedules were administered to all participants. As shown in **Table 2**, baseline comparisons across the three intervention groups demonstrated no statistically significant differences in CARS total scores or in any subscale of the Gesell Developmental Schedules (all P > 0.05), indicating that the groups were comparable at baseline. These same assessments were then re-administered immediately upon completion of the 6-month intervention period to evaluate post-intervention outcomes.

**Table 2:** Baseline comparison of CARS and Gesell dimensional DQ scores among the three groups of children before intervention.

Parameter	Institutional intervention	Family intervention	Combined family-institutional intervention	*H*	*P*-value
CARS	34.00 (29.00, 39.00)	34.00 (28.50, 42.00)	34.75 (29.50, 40.00)	0.140	0.932
Adaptability	85.00 (42.00, 112.00)	84.00 (52.00, 109.00)	82.00 (44.00, 108.00)	0.857	0.651
Gross motor	93.00 (51.00, 109.00)	93.00 (57.00, 112.00)	87.50 (67.00, 113.00)	5.880	0.053
Fine motor	91.00 (57.00, 119.00)	90.00 (57.00, 143.00)	83.00 (50.00, 129.00)	5.503	0.064
Language	66.00 (30.00, 80.00)	59.00 (31.00, 86.00)	57.50 (32.00, 80.00)	5.414	0.067
Personal-social	74.00 (42.00, 89.00)	77.00 (49.00, 112.00)	76.50 (43.00, 105.00)	4.520	0.104

Data are presented as median (interquartile range). The comparison between the three groups were assessed using the Kruskal-Wallis test (Z values). P values are two-tailed. ^***^p < 0.001, ^**^p < 0.01, ^*^p < 0.05.

### 2.2 Measures

Assessments were administered at baseline and immediately post-intervention (6 months) by two senior pediatricians blinded to group allocation.

#### 2.2.1 Autism symptoms

The primary outcome was the change in autism symptom severity as measured by the Childhood Autism Rating Scale (CARS). Although primarily diagnostic, CARS is validated for quantifying severity changes in young children. To ensure consistency, the same blinded assessor evaluated each child at both time points. Severity was classified as mild-moderate (CARS < 36) or severe (CARS≥36) (14).

#### 2.2.2 Developmental status

The Chinese revised version of the Gesell Developmental Schedules (15, 16) was used to assess the Developmental Quotient (DQ). This scale covers five domains: adaptability, gross motor, fine motor, language, and personal-social skills. A DQ < 75 indicates developmental delay.

#### 2.2.3 Quality control

All therapists participating in the trial underwent standardized training to achieve proficiency in Applied Behavior Analysis (ABA), sensory integration therapy, cognitive training, language therapy, and the Early Start Denver Model (ESDM). Mandatory assessment was required to certify their qualifications in Autism Spectrum Disorder (ASD) intervention. All assessors held health technology qualification certificates and underwent unified training to ensure consistent understanding and application of the scales. All assessors were blinded to the children’s group allocation, and pre- and post-intervention assessments for the same child were conducted by the same assessor. Rehabilitation training was conducted in formal children’s health care centers. General information was collected immediately after obtaining parental consent using standardized forms. Data entry was performed independently by two professionals followed by cross-validation. Safety measures were strictly implemented during all sensory integration and game courses.

#### 2.2.4 Statistical analysis

Primary Outcome: The change in the total score of the Childhood Autism Rating Scale (CARS) from baseline to the end of the intervention period was prespecified as the sole primary outcome to assess the severity of core ASD symptoms. Sample size calculations were based exclusively on this endpoint. Secondary Outcomes: We have clarified that the domains of the Gesell Developmental Schedule (GDS) (including gross motor, fine motor, adaptive behavior, language, and personal-social) are designated as secondary outcomes. These were analyzed to evaluate broader developmental changes.

Data entry was performed using Microsoft Excel, with quality control ensured through double independent entry and cross-validation. All statistical analyses were conducted using SPSS version 25.0. The normality of continuous variables was assessed using the Shapiro–Wilk test. Given the inherent heterogeneity of the autism spectrum disorder (ASD) population, most outcome variables deviated from a normal distribution; consequently, continuous data are summarized as median and interquartile range [M(P25,P75)].

For overall comparisons across the three study groups, the Kruskal–Wallis H test was employed. Within-group changes from baseline to post-intervention were evaluated using the Wilcoxon signed-rank test. Effect sizes for these paired comparisons were quantified using the rank-biserial correlation coefficient (r_rb_), calculated as r_rb_=1−2W/n(n+1), where W is the sum of positive ranks and n is the number of non-zero differences.

To compare post-intervention outcomes between intervention groups while adjusting for baseline differences, analysis of covariance (ANCOVA) models were fitted. In each ANCOVA model, the post-intervention score of the specific outcome served as the dependent variable, and the intervention group served as the independent variable. Covariate selection was prespecified based on clinical relevance and potential confounding effects, rather than solely on baseline statistical significance. Accordingly, each model included the corresponding baseline score of the outcome variable as a mandatory covariate. Additionally, the following demographic and perinatal variables were simultaneously included as covariates in all models: age, sex, place of residence, singleton status, parental age at childbirth, parental education level, birth order, parity, gestational age at delivery, and mode of delivery. Robust standard errors (HC3) were applied to all ANCOVA models to account for potential heteroscedasticity. Model assumptions, including linearity, homogeneity of regression slopes, and normality of residuals, were assessed via residual plots and statistical tests; no significant violations were observed. Overall group effects were assessed using Wald χ² statistics, followed by Holm-adjusted *post hoc* pairwise comparisons where applicable.

To examine whether baseline ASD severity modified intervention effects, additional ANCOVA models incorporating a group × severity interaction term were constructed, with interaction effects evaluated via Wald χ² tests. Stratified analyses by baseline ASD severity were performed for descriptive purposes only, with within-group pre–post changes again assessed using the Wilcoxon signed-rank test.

Regarding the interpretation of standardized Z-values presented in the results **Tables 3**–**5**: For the CARS, where lower scores indicate fewer symptoms, a positive Z-value reflects a greater reduction in symptom severity (i.e., improvement) in the intervention group relative to the reference. Conversely, for Gesell Developmental Schedule domains, where higher scores indicate better performance, a positive Z-value reflects greater improvement in developmental quotients. To ensure consistency and clarity, all Z-values in the revised tables have been standardized such that a positive value universally indicates a favorable treatment effect (i.e., symptom reduction for CARS and performance enhancement for Gesell domains). Any negative Z-values therefore indicate a lesser treatment effect compared to the reference group.

**Table 3:** Within-group pre–post changes in CARS scores and adjusted between-group comparisons among intervention groups.

Group	Pre-intervention	Post-intervention	*Z*	Effect size (r)	Wald χ²	Holm-adjusted post hoc
Institutional intervention^1^	34.00 (29.00, 39.00)	33.00 (28.00, 38.00)	4.14^***^	0.62	9.96^**^	3-1: adjusted difference=-1.19 (95% CI -1.97 to -0.40), Holm P = 0.011; 2–1 and 3-2, no significant pairwise difference.
Family intervention^2^	34.00 (28.50, 42.00)	31.00 (27.00, 38.00)	5.59^***^	0.83		
Combined family-institutional intervention^3^	34.75 (29.50, 40.00)	32.50 (28.00, 36.00)	6.33^***^	0.98		

Data are presented as median (interquartile range). Within-group changes were assessed using Wilcoxon signed-rank tests (Z values, positive Z-values consistently represent clinical improvement, i.e., reduced CARS scores), with effect sizes reported as rank-biserial correlation (r). Between-group differences were examined using ANCOVA adjusting for baseline CARS and covariates, with Wald χ² statistics reported. *Post hoc* pairwise comparisons were adjusted using the Holm method. P values are two-tailed. ^***^p < 0.001, ^**^p < 0.01, ^*^p < 0.05.

^1^institutional intervention (Group 1), ^2^family intervention (Group 2), ^3^combined family-institutional intervention (Group 3).

**Table 4:** Within-group pre–post changes in Gesell developmental quotients and adjusted between-group comparisons among intervention groups.

Parameter	Pre-intervention	Post-intervention	*Z*	Effect size (r)	Wald χ²	Holm-adjusted post hoc
Adaptability							
Institutional intervention^1^	85.00 (42.00, 112.00)	87.00 (58.00, 110.00)	2.63^**^	0.47	3.26	No significant pairwise difference
Family intervention^2^	84.00 (52.00, 109.00)	85.00 (60.00, 105.00)	2.65^**^	0.45		
Combined family-institutional intervention^3^	82.00 (44.00, 108.00)	83.50 (71.00, 118.00)	4.42^***^	0.74		
Gross motor							
Institutional intervention^1^	93.00 (51.00, 109.00)	95.00 (49.00, 110.00)	0.71	0.11	2.21	No significant pairwise difference
Family intervention^2^	93.00 (57.00, 112.00)	90.00 (71.00, 113.00)	-1.20	0.18
Combined family-institutional intervention^3^	87.50 (67.00, 113.00)	85.00 (63.00, 116.00)	-1.03	0.18
Fine motor							
Institutional intervention^1^	91.00 (57.00, 119.00)	90.00 (41.00, 120.00)	-0.80	0.14	6.13^*^	3-1: adjusted difference=4.88 (95% CI 1.34 to 8.43), Holm P = 0.021; 2–1 and 3–2 no significant pairwise difference.
Family intervention^2^	90.00 (57.00, 143.00)	93.00 (48.00, 135.00)	2.06^*^	0.35
Combined family-institutional intervention^3^	83.00 (50.00, 129.00)	88.50 (62.00, 125.00)	4.42^***^	0.73
Language							
Institutional intervention^1^	66.00 (30.00, 80.00)	70.00 (39.00, 104.00)	4.30^***^	0.64	8.61^*^	3-2: adjusted difference=6.35 (95% CI 2.37 to 10.32), Holm P = 0.015; 2–1 and 3–1 no significant pairwise difference.
Family intervention^2^	59.00 (31.00, 86.00)	72.00 (38.00, 105.00)	6.45^***^	0.96
Combined family-institutional intervention^3^	57.50 (32.00, 80.00)	72.00 (50.00, 98.00)	6.71^***^	0.99
Personal-social							
Institutional intervention^1^	74.00 (42.00, 89.00)	75.00 (50.00, 92.00)	1.73	0.31	6.37^*^	2-1: adjusted difference=3.93 (95% CI 0.97 to 6.89), Holm P = 0.028; 2–3 and 3-1, no significant pairwise difference.
Family intervention^2^	77.00 (49.00, 112.00)	85.00 (59.00, 115.00)	4.01^***^	0.71
Combined family-institutional intervention^3^	76.50 (43.00, 105.00)	78.00 (58.00, 100.00)	3.27^**^	0.56

Data are presented as median (interquartile range). Within-group changes were assessed using Wilcoxon signed-rank tests (Z values, positive Z-values consistently represent clinical improvement, i.e., reduced CARS scores or increased Gesell DQ scores), with effect sizes reported as rank-biserial correlation (r). Between-group differences were examined using ANCOVA adjusting for baseline developmental quotients and covariates, with Wald χ² statistics reported. *Post hoc* pairwise comparisons were adjusted using the Holm method. P values are two-tailed. ^***^p < 0.001, ^**^p < 0.01, ^*^p < 0.05.

^1^institutional intervention (Group 1), ^2^family intervention (Group 2), ^3^combined family-institutional intervention (Group 3).

**Table 5:** Pre–post changes in CARS and Gesell developmental quotients stratified by baseline ASD severity.

Parameter	Mild-moderate (*N* = 74)		*Z*	Severe (*N* = 58)		*Z*	Group × severity
	Pre-intervention	Post-intervention		Pre-intervention	Post-intervention		
CARS							
Institutional intervention	32.00 (31.00–34.00)	32.00(31.00–33.00)	1.00	38.00 (37.00–38.63)	36.50 (32.75–37.25)	3.89^***^	3.00
Family intervention	32.00 (31.00–33.75)	30.50 (30.00–31.00)	3.02^**^	38.00(37.00–38.50)	36.00 (35.00–37.00)	4.28^***^	
Combined family-institutional intervention	33.00 (32.00–34.00)	30.00 (30.00–31.00)	4.41^***^	37.00 (36.50–38.50)	35.00 (34.00–35.00)	3.75^***^	
Adaptability							
Institutional intervention	80.00 (66.00–96.00)	83.00 (70.00–95.00)	1.45	86.50 (79.25–93.00)	88.50 (73.50–99.25)	2.22^*^	1.63
Family intervention	83.00 (73.00–93.28)	86.50 (78.25–95.75)	1.67	84.00 (70.00–95.50)	85.00 (77.50–96.00)	2.01^*^	
Combined family-institutional intervention	76.00 (70.00–83.50)	83.00 (76.50–87.00)	3.03^**^	88.00 (81.50–92.50)	90.00 (81.00–98.50)	2.93^**^	
Gross motor							
Institutional intervention	92.00 (87.00–96.00)	90.00 (82.00–98.00)	0.02	94.00 (87.75–99.25)	96.50 (82.25–101.0)	0.99	0.47
Family intervention	92.00 (85.25–97.75)	89.50 (86.00–96.75)	0.00	89.00 (83.00–102.0)	96.00 (89.00–98.50)	2.01^*^	
Combined family-institutional intervention	87.00 (80.50–89.50)	82.00 (76.50–89.00)	-1.64	89.00 (85.50–93.00)	93.00 (84.00–98.50)	0.53	
Fine motor							
Institutional intervention	90.00 (78.00–97.00)	89.00 (77.00–97.00)	0.53	95.00 (87.00–98.50)	94.00 (83.25–99.25)	0.53	0.81
Family intervention	91.00 (73.75–99.75)	97.00 (82.25–100.0)	1.34	88.00 (72.00–96.50)	90.00 (78.00–96.50)	1.50	
Combined family-institutional intervention	80.00 (72.50–89.00)	86.00 (81.00–94.50)	3.92^***^	84.00 (75.50–96.00)	91.00 (82.00–99.50)	2.14^*^	
Language							
Institutional intervention	62.00 (57.00–70.00)	68.00 (63.00–75.00)	3.05^**^	69.00 (62.75–71.50)	70.00 (65.00–77.25)	2.09^*^	2.84
Family intervention	58.00 (51.50–68.93)	74.00 (65.75–79.75)	5.12^***^	64.00 (49.00–72.50)	66.00 (57.50–78.50)	4.04^***^	
Combined family-institutional intervention	57.00 (48.00–61.00)	71.00 (64.00–79.50)	5.17^***^	60.00 (55.50–68.50)	73.00 (64.00–77.50)	4.39^***^	
Personal-social							
Institutional intervention	74.00 (70.00–78.00)	76.00 (72.00–83.00)	2.64^**^	75.00 (67.75–78.25)	73.50 (67.75–79.25)	0.04	0.50
Family intervention	76.50 (70.75–82.00)	85.50 (74.00–89.00)	3.30^***^	79.00 (68.50–86.00)	78.00 (70.00–89.00)	2.21^*^	
Combined family-institutional intervention	77.00 (67.50–79.50)	78.00 (68.50–87.50)	2.57^**^	76.00 (67.00–79.50)	78.00 (70.00–83.50)	1.92	

Data are presented as median (interquartile range). Within-group changes were assessed using Wilcoxon signed-rank tests ((Z values, positive Z-values consistently represent clinical improvement, i.e., reduced CARS scores or increased Gesell DQ scores). Group × severity interaction effects were examined using ANCOVA adjusted for baseline values and covariates, with Wald χ² statistics reported. P values are two-tailed. ***p < 0.001, **p < 0.01, *p < 0.05.

All statistical tests were two-tailed, and a P < 0.05 was considered statistically significant. Furthermore, to control for type I error inflation arising from multiple comparisons across secondary outcome domains, the Bonferroni correction was applied. While these secondary outcomes provide important contextual data, the main conclusions of this study rely primarily on the prespecified primary outcome.

## 3 Results

### 3.1 Comparison of CARS scores across intervention groups

Within-group analyses showed that CARS scores decreased significantly after intervention in all three groups, including the institutional intervention, family intervention, and combined family-institutional intervention groups (all P < 0.001; **Table 3**).

After adjustment for baseline CARS scores and relevant covariates using ANCOVA, a significant overall group effect was observed (Wald χ² = 9.96, P < 0.01). *Post hoc* pairwise comparisons with Holm adjustment indicated that the combined family-institutional intervention group had lower post-intervention CARS scores than the institutional intervention group (P < 0.05). No statistically significant differences were observed between the family and institutional intervention groups or between the family and combined intervention groups (**Table 3**).

### 3.2 Comparison of Gesell developmental quotients across intervention groups within-group changes

Wilcoxon signed-rank tests demonstrated significant within-group improvements across multiple Gesell domains, with domain-specific patterns observed among intervention groups (**Table 4**). In the institutional intervention group, significant improvements were observed in the adaptability and language domains (P < 0.05), whereas no significant changes were detected in gross motor, fine motor, or personal–social domains. In the family intervention group, significant improvements were observed in the adaptability, fine motor, language, and personal-social domains (all P < 0.05). In the combined family-institutional intervention group, significant improvements were observed in the adaptability, fine motor, language, and personal–social domains (all P < 0.05).

### 3.3 Between-group comparisons

After adjustment for baseline developmental quotients and covariates using ANCOVA, significant between-group differences were observed in selected Gesell domains (**Table 4**). A significant overall group effect was detected for the fine motor domain (Wald χ² = 6.13, P < 0.05). *Post hoc* analyses revealed that the combined family-institutional intervention group demonstrated significantly higher post-intervention fine motor scores compared with the institutional intervention group after Holm correction (P < 0.05). For the language domain, the overall group effect was also significant (Wald χ² = 8.61, P < 0.05). Holm-adjusted pairwise comparisons indicated that the combined family-institutional intervention group outperformed the institutional intervention group (P < 0.05), whereas no other pairwise comparisons reached statistical significance. In the personal-social domain, a significant overall group effect was observed (Wald χ² = 6.37, P < 0.05). *Post hoc* comparisons showed that the family intervention group had significantly higher post-intervention personal-social scores than the institutional intervention group after Holm adjustment (P < 0.05). No significant differences were observed between the combined intervention group and the other two groups. No statistically significant between-group differences were found in the adaptability or gross motor domains (all P > 0.05).

### 3.4 Severity-stratified pre–post changes in CARS and Gesell developmental quotients

Regarding the baseline severity distribution, there were no significant differences in the proportion of children with mild-moderate versus severe ASD across the three intervention groups (χ^2^=0.087, P = 0.957). Specifically, in the institution intervention group, 25 children (55.6%) were classified as mild-moderate and 20 (44.4%) as severe. In the family intervention group, 26 (19%) presented with mild-moderate symptoms and 19 (42.2%) with severe symptoms. Similarly, the combined family-institution group comprised 23(54.8%) children with mild-moderate ASD and 19 (45.2%) with severe ASD (**Table 1**). This balanced distribution ensures that subsequent comparisons of intervention efficacy are not confounded by baseline severity disparities.

Severity-stratified descriptive analyses of pre–post changes in CARS scores and Gesell developmental quotients are presented in **Table 5**. Across both mild–moderate and severe ASD subgroups, CARS scores decreased significantly after intervention in all three intervention groups, including institutional, family, and combined family–institutional interventions (all P < 0.05).

For the Gesell Developmental Scale, domain-specific patterns of within-group improvement were observed across severity strata.

In the mild–moderate ASD subgroup, significant post-intervention improvements were observed in the language domain across all three intervention groups (all P < 0.01). Improvements in adaptability were significant in the combined family–institutional intervention group, whereas improvements in fine motor and personal–social domains were primarily observed in the family and combined intervention groups. No consistent significant changes were observed in the gross motor domain across intervention groups.

In the severe ASD subgroup, significant improvements were again observed in CARS scores across all intervention groups (all P < 0.01). Improvements in the language domain were evident across all three intervention groups, whereas significant gains in adaptability, fine motor, and personal–social domains were mainly observed in the family intervention and combined family-institutional intervention groups. Changes in the gross motor domain were limited and inconsistent across intervention groups.

Formal testing of effect modification using ANCOVA models with a group × severity interaction term revealed no statistically significant interaction effects for CARS scores or any Gesell developmental domain (all P > 0.05), indicating that the overall pattern of intervention effects was not significantly modified by baseline ASD severity.

## 4 Discussion

This study evaluated three distinct intervention models for children with Autism Spectrum Disorder (ASD) over a six-month period. All models demonstrated improvements in both ASD symptoms and developmental abilities. Notably, family intervention yielded significantly greater improvements in personal-social skills compared to institution intervention. The combined intervention was associated with greater improvement in selected outcomes after adjustment for measured covariates, demonstrating more pronounced benefits in reducing core ASD symptoms and enhancing fine motor and language abilities compared to single-setting approaches.

Stratified analyses revealed descriptive trends in domain-specific improvements across baseline severity levels, though we acknowledge that the group × severity interaction effects were not statistically significant and thus these findings should be interpreted as exploratory. Specifically, while children with both mild–moderate and severe ASD demonstrated significant post-intervention improvements in language (and CARS scores for the severe subgroup) across all models, greater gains in adaptability, fine motor, and personal-social domains were primarily observed in the family or combined family-institutional intervention groups. Consequently, rather than claiming severity-specific superiority, our primary conclusion emphasizes that, after adjustment for measured covariates, the combined family-institutional intervention was associated with more comprehensive improvements in adaptive and fine motor domains compared to other models across the overall cohort; however, these observations remain limited by the non-randomized allocation and insufficient statistical power to detect interaction effects.

These findings align with a substantial body of research consistently demonstrating that early intervention for infants and toddlers with ASD is effective in improving core symptoms, cognition, language, social communication, and adaptive behaviors. Sandbank et al. (17) reported that behavioral interventions yield significant, clinically meaningful improvements in cognition, language, social communication, and adaptive behavior, highlighting intervention intensity (hours per week) and younger age at initiation as critical moderating factors. French & Kennedy (18) also strongly affirm the efficacy of early intervention for improving ASD core symptoms (social communication, repetitive behaviors) and overall development, discussing the evidence base for various models (e.g., ESDM, JASPER, PACT). Furthermore, both international and national guidelines emphasize the critical importance of early intervention. The American Academy of Pediatrics (19)strongly recommends initiating evidence-based intervention as early as possible for all children diagnosed with or at high likelihood of ASD, a stance echoed in China’s ASD rehabilitation guidelines since 2010 (20). Therefore, early intervention demonstrably enhances developmental abilities and ameliorates symptoms in children with ASD.

This study underscores the significant value of family intervention models, corroborating existing research. Within family programs, parents/caregivers can apply skills learned from professionals to real-life settings, potentially enhancing the effects of other interventions (21). Our family intervention model, guided by principles of the Early Start Denver Model (ESDM), proved effective in enhancing language (22), Personal-social (23) and fine motor skills (24). Consistent with recommendations that family intervention requires professional guidance, our family intervention group showed significant improvements in language abilities and ASD symptoms after six months. Studies suggest that over longer periods (e.g., one year), family intervention may lead to more substantial gains in language and fine motor skills compared to conventional care (25).This study provides an initial exploration comparing the effectiveness of institution, intervention family intervention, and combined family-institution intervention models for children with ASD. The results indicate that the combined family-institution intervention model offers comprehensive benefits, demonstrating statistically significant advantages in reducing ASD symptoms and improving key developmental domains like fine motor and language skills compared to the single-setting approaches.

The more pronounced benefits of the combined family-institutional model in reducing core ASD symptoms and enhancing fine motor and language abilities are consistent with the growing recognition of the importance of cross-contextual intervention delivery. The PACT-G trial, which delivered intervention in parallel at home and in education settings, demonstrated that while the primary outcome did not show a significant treatment effect, the intervention preserved positive proximal outcomes and improved parental wellbeing and child disruptive behaviors across home and school contexts (26). This highlights the potential of multi-context delivery to address the challenge of generalization beyond the immediate treatment setting, even if the magnitude of effect on core symptom measures may vary depending on dosage, delivery mode, and contextual challenges (27). Similarly, a hospital-family collaborative DTT program showed significant improvements in children’s developmental outcomes and family functioning, with partial correlation analyses suggesting that improvements in parenting stress were linked to changes in adaptive and behavioral items during the hospital and family phases, respectively (28). This supports the notion that sustained family intervention, supported by professional guidance, can contribute meaningfully to both child outcomes and family dynamics (29).

The differential responses observed in our study—where the combined model showed particular advantages in language and fine motor skills, and where the benefits of family and combined interventions were more pronounced in mild-to-moderate ASD—can be interpreted through the lens of intervention generalization and dosage. Parent-mediated approaches are appealing because they allow intervention to occur in naturalistic contexts integrated into daily routines, thereby increasing the effective “dose” of intervention and promoting generalization and maintenance of acquired skills (30). A systematic review and transfer analysis (31) further emphasized that interventions delivered collaboratively by both parents and clinicians showed the largest effects on spoken language outcomes, followed by parent-only and clinician-only delivery. This aligns with our finding that the combined model, which leverages both institutional expertise and family implementation, produced greater gains in language and fine motor skills. Moreover, the observation that language gains were greater in mild-to-moderate ASD within the family and combined groups may reflect a threshold effect, where children with less severe baseline impairments are better positioned to capitalize on the increased opportunities for practice and generalization afforded by family involvement.

It is important to acknowledge that the evidence base for multi-context delivery is nuanced. The PACT-G trial (26) serves as a cautionary example that simply changing delivery methods and contexts does not automatically preserve the efficacy of an original intervention, potentially due to reduced component doses, remote delivery, and contextual challenges. Nevertheless, the consistent theme across studies is that involving parents in interventions reinforces the effectiveness of the intervention and promotes skill generalization outside of the school or clinic setting (32, 33). Family–school partnerships and parent involvement models have been associated with improvements in social communication and reductions in restrictive and repetitive behaviors in children with ASD (34). In our study, the combined model appears to harness these advantages by integrating professional guidance with family implementation, leading to broader developmental gains.

### 4.1 Limitations

Several limitations warrant consideration. Several limitations warrant consideration. First, this study employed a non-randomized design. The allocation of participants to the three intervention groups was based on parental preference and clinical availability rather than random assignment. This introduces the potential for selection bias, as families who chose the combined or family intervention models might differ systematically from those choosing institution intervention care in terms of socioeconomic status, parental education, or motivation, which could confound the observed outcomes. Second, the relatively small sample size (n=132) may not fully capture the heterogeneity of the ASD population, potentially limiting the generalizability of the findings. Third, Although the prescribed total weekly intervention duration was uniformly 25 hours, the three groups differed in setting, delivery format, caregiver and therapist involvement, and implementation fidelity. Thus, outcome differences cannot be attributed solely to the intervention model. Unfortunately, data were insufficient to quantify intergroup differences in actual completed hours, attendance, video submission, or adherence. Future studies should collect these metrics at the design stage to better assess true intervention exposure and its impact on outcomes. Fourth, although all assessments were conducted by trained evaluators blinded to group allocation, and the same assessor evaluated each child before and after the intervention to ensure consistency, inter-rater reliability was not formally assessed. This limitation should be acknowledged and addressed in future research to enhance the robustness and reproducibility of the scoring process. Fifth, the six-month intervention period was insufficient for evaluating long-term developmental trajectories and the maintenance of gains; future research should prioritize larger cohorts with extended follow-up durations. Sixth, this study had an attrition rate of 12% (18/150). Consequently, the possibility of attrition bias cannot be entirely ruled out, particularly given the small sample size of the non-completer group. Future studies with larger samples should employ intention-to-treat (ITT) analysis to mitigate this potential bias. Finally, the lack of statistical power to detect interaction effects limits our ability to draw firm conclusions about severity-specific superiority. The group × severity interaction terms were non-significant, and our stratified analyses should be regarded as descriptive and hypothesis-generating rather than confirmatory.

## 5 Conclusion

In conclusion, our findings support the integration of family intervention components into ASD intervention frameworks, particularly when combined with institutional support. The combined family-institutional model emerges as a promising approach for achieving comprehensive developmental benefits, especially in language and fine motor skills. Future research should focus on optimizing the balance between institutional and family intervention components, ensuring fidelity of delivery across contexts, controlling for total intervention intensity, and extending follow-up periods to assess the long-term maintenance of gains. Additionally, given the variability in response based on baseline severity, personalized intervention planning that considers the child’s symptom profile and developmental level is essential for maximizing outcomes.

## Data Availability

The original contributions presented in the study are included in the article/supplementary material, further inquiries can be directed to the corresponding author/s.
